# Ludwig’s Angina Complicated by a Massive Bilateral Deep Neck Abscess in a 26-Month-Old Boy: Multidisciplinary Airway and Surgical Management

**DOI:** 10.7759/cureus.117232

**Published:** 2026-09-30

**Authors:** Razan Ismail, Khadejah Jamal, Rehaam Fathima, Safeena Kherani

**Affiliations:** 1 Pediatric Emergency Medicine, Al Jalila Children's Speciality Hospital, Dubai, ARE; 2 Ear, Nose, and Throat, Al Jalila Children's Speciality Hospital, Dubai, ARE; 3 Pediatric Emergency Medicine, Dubai Medical University, Dubai, ARE; 4 Otolaryngology - Head and Neck Surgery, Mohammed Bin Rashid University, Dubai, ARE; 5 Otolaryngology - Head and Neck Surgery, Al Jalila Children's Speciality Hospital, Dubai, ARE

**Keywords:** airway management, case report, ludwig’s angina, pediatric deep neck infection, staphylococcus aureus, sublingual abscess, submandibular abscess

## Abstract

Ludwig’s angina is a rapidly progressive deep neck infection affecting the submandibular, sublingual, and submental regions. Although uncommon in children, it can be life-threatening due to sudden airway compromise. Early diagnosis, multidisciplinary airway planning, broad-spectrum antibiotic therapy, and timely surgical source control are essential in pediatric cases.

We report a previously healthy, unimmunized 26-month-old boy who presented with acute fever, rapidly progressive bilateral submandibular and submental neck swelling, poor feeding, vomiting, diarrhea, lethargy, inability to lie flat, and restricted neck movement. No drooling or stridor was present at presentation. Examination revealed massive fluctuant swelling throughout the submandibular and submental regions. Initial ultrasound demonstrated an extensive echogenic collection measuring approximately 15 × 4 cm, extending from the left submandibular region across the submental space to the right submandibular region. Contrast-enhanced CT demonstrated a large bilateral submandibular and submental abscess extending into the sublingual space and floor of the mouth, with mild airway mass effect but no intracranial extension. Due to the high risk of airway compromise, elective intubation was performed in the PICU before CT imaging and surgery. Urgent incision and drainage of the extensive bilateral submandibular, submental, and sublingual deep neck abscess was performed, yielding approximately 150 mL of purulent material, and a Penrose drain was placed. Two wound cultures grew methicillin-sensitive *Staphylococcus aureus*, and antimicrobial therapy was subsequently narrowed to flucloxacillin based on susceptibility results. After successful extubation, weaning to room air, and tolerating oral intake, the patient was discharged in stable condition to complete a 14-day course of antibiotic therapy.

This case highlights that pediatric Ludwig’s angina can progress rapidly and pose a significant airway risk even without drooling or stridor. Early multidisciplinary involvement, controlled airway management, cross-sectional imaging, prompt surgical drainage, and culture-directed antimicrobial therapy are crucial for a favorable outcome. A high index of suspicion and early airway planning are particularly important in children with rapidly progressive deep neck infections.

## Introduction

Ludwig's angina is an infection of the floor of the mouth and the deep neck spaces, commonly affecting the submandibular, sublingual, and submental regions [1-3]. Although Ludwig’s angina is classically characterized by rapidly spreading cellulitis of the submandibular and sublingual spaces, it can progress to a localized or extensive deep neck abscess. In such cases, distinguishing the underlying Ludwig-space infection from the resulting abscess formation is important when describing the clinical and radiological findings. As a consequence of pediatric deep neck infections, airway obstruction, sepsis, and mediastinal spread are known to be life-threatening complications [2,3].

Early recognition, thorough airway assessment, and broad-spectrum intravenous antibiotics are essential, while imaging is performed when safe to determine disease extent. Surgical drainage is indicated when an abscess forms or clinical deterioration occurs [4]. A particular challenge in pediatric airway management is the difficulty of performing awake fiberoptic techniques in young children. Intubation should be performed under the supervision of senior anesthesia and otolaryngology personnel in a controlled environment [5].

Our report presents a 26-month-old boy with Ludwig’s angina complicated by extensive bilateral deep neck abscess formation and significant airway risk, requiring elective intubation, urgent incision and drainage, and culture-directed antimicrobial therapy for methicillin-sensitive *Staphylococcus aureus* (MSSA) infection. This case highlights the potential for rapid progression and significant airway compromise in pediatric Ludwig’s angina, even without classical features such as drooling or stridor, and emphasizes the importance of early airway assessment, multidisciplinary management, prompt imaging, and timely surgical source control. This case report was prepared in accordance with the CARE case report guidelines [5].

## Case presentation

A previously healthy, unimmunized 26-month-old boy presented to the ED with a one-day history of fever and rapidly progressive neck swelling. His symptoms were associated with vomiting, two episodes of loose stools, poor feeding, lethargy, inability to lie flat, and reduced tolerance of solid food, although he continued to tolerate liquids. There was no history of rash, drooling, stridor, urinary symptoms, recent travel, recent antibiotic exposure, or known contact with an ill individual.

On arrival, the child was irritable but hemodynamically stable. His initial vital signs were a heart rate of 120 beats/min, a respiratory rate of 26 breaths/min, an oxygen saturation of 99% on room air, a temperature of 36.6°C following a reported fever of 38.5°C, a blood pressure of 85/35 mmHg, and a weight of 14 kg. A complete oral examination was deferred in the ED and remained limited in the PICU due to concern for airway compromise; tongue-depressor instrumentation was avoided until intubation. No definite odontogenic source was identified. Examination showed extensive fluctuant bilateral submandibular and submental swelling crossing the midline, a soft, boggy floor of mouth, and restricted neck movement, without drooling or stridor.

Initial laboratory investigations demonstrated evidence of systemic inflammation, including an elevated C-reactive protein of 87.6 mg/L (reference range: 0.4-1.3 mg/L) and an erythrocyte sedimentation rate of 118 mm/h (reference range: 0-20 mm/h). The procalcitonin level subsequently increased during admission before declining with treatment. The admission full blood count showed leukocytosis (WBC 15.9 × 10³/µL; reference range: 5-15 × 10³/µL) and thrombocytosis (platelets 600 × 10³/µL; reference range: 200-490 × 10³/µL), with microcytic indices consistent with iron-deficiency anemia. Given the rapidly progressive neck swelling, restricted neck movement, inability to lie flat, and concern for impending airway compromise despite the absence of stridor or drooling, the patient was referred for urgent multidisciplinary assessment and airway planning.

A bedside neck ultrasound revealed an extensive, thick, echogenic collection measuring approximately 15 cm in width and 4 cm in depth. The collection extended across the left and right submandibular regions and the submental region. Ultrasound showed no apparent deeper extension, and the carotid vessels, thyroid gland, and parotid glands appeared normal; the overlying collection obscured the submandibular glands.

Following multidisciplinary assessment and securing the airway with elective endotracheal intubation, a contrast-enhanced CT of the neck was performed to assess for airway compromise or deep neck extension. CT demonstrated a large rim-enhancing abscess measuring approximately 9.6 × 4.8 × 6.3 cm. The abscess involved the bilateral submandibular and submental spaces; extended superiorly into the sublingual space and floor of the mouth; inferiorly into the lower cervical fascial plane; posteriorly toward the parapharyngeal space up to the hyoid level; and laterally into the cervical fascial planes. A mild mass effect on the airway was noted. Brain CT showed no acute intracranial abnormality.

Chest imaging obtained during the PICU course showed right-sided atelectatic changes. The patient's respiratory status subsequently improved with supportive care. Figure 1 shows the initial ultrasound and subsequent contrast-enhanced CT findings.

**Figure 1:**
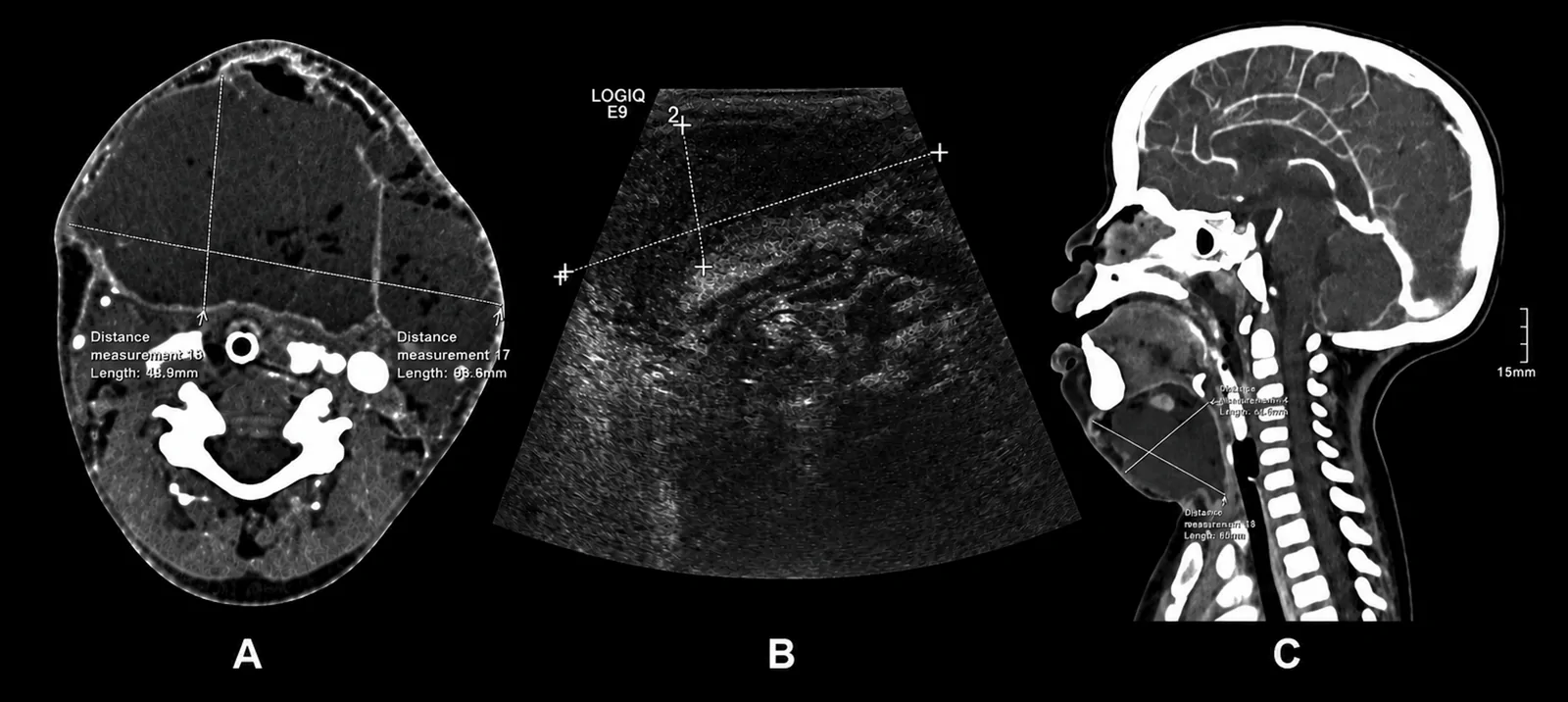
Imaging findings at presentation (A) Axial contrast-enhanced CT image of the neck reveals a large rim-enhancing collection involving the bilateral submandibular and submental spaces with extension toward the sublingual space and floor of the mouth and mild mass effect on the airway. (B) Soft-tissue ultrasound image showing an extensive echogenic collection across the submandibular/submental region. These imaging findings were consistent with Ludwig’s angina. (C) Sagittal contrast-enhanced CT image demonstrating the extent of the collection in the submandibular/submental spaces with superior extension toward the sublingual space and mass effect on the airway. CT: computed tomography

Therapeutic intervention

The child initially received intravenous vancomycin (15 mg/kg/dose, administered within one hour of arrival) and piperacillin-tazobactam (100 mg/kg/dose, administered within one hour of arrival) in the ED as empiric broad-spectrum antimicrobial therapy. Table 1 summarizes the overall clinical course, including airway management, imaging, surgical drainage, postoperative care, and subsequent antimicrobial de-escalation.

**Table 1:** Management course of a 26-month-old boy with deep neck space abscess ED: emergency department, PICU: pediatric intensive care unit, ENT: ear, nose, and throat, ID: infectious diseases, CT: computed tomography, CRP: C-reactive protein, ESR: erythrocyte sedimentation rate, WBC: white blood cell, MSSA: methicillin-sensitive *Staphylococcus aureus*

Time point	Clinical status/key findings	Investigations	Management/interventions	Outcome
0 h on arrival (ED)	One-day history of fever and rapidly progressive neck swelling. Vomiting, loose stools, poor feeding, lethargy. Unable to lie flat, reduced intake of solids. No drooling or stridor. Hemodynamically stable.	Labs: ↑CRP 87.6 mg/L, ESR 118 mm/h, ↑procalcitonin. WBC 15.9 × 10³/µL, platelets 600 × 10³/µL, microcytic anemia. Sepsis workup sent.	IV vancomycin 15 mg/kg (within 1 h). IV piperacillin-tazobactam 100 mg/kg/dose (on arrival). Early involvement of ENT, pediatric surgery, anesthesia, radiology, ID, PICU teams. Dexamethasone deferred.	Stabilized and monitored. High risk of airway compromise identified.
5 h after presentation (PICU)	Anticipated difficult airway. Need for safe transfer for CT imaging.	-	Elective endotracheal intubation. Difficult-airway trolley prepared. Video laryngoscopy in ramped position with anesthesia and ENT support. First-pass successful. Sedation and mechanical ventilation continued.	Hemodynamically uneventful; airway secured.
After intubation	Extent of deep neck infection and airway involvement needs assessment.	Contrast-enhanced CT neck: large rim-enhancing abscess (~9.6 × 4.8 × 6.3 cm) involving bilateral submandibular, submental, sublingual spaces; extending to floor of mouth; inferiorly to lower cervical fascial plane; posteriorly to parapharyngeal space (up to hyoid level); laterally into cervical fascial planes. Mild mass effect on airway. CT brain: no acute intracranial abnormality.	- (Imaging to guide intervention.)	Extent defined; no intracranial involvement.
6 h after presentation (within 30 min of CT)	Extensive deep neck abscess confirmed. Requires definitive source control.	-	Urgent incision and drainage. ~150 mL purulent fluid with debris drained. Large left submandibular pocket crossing midline via sublingual space to right submandibular space. Irrigation, cultures taken. 8-cm Penrose drain + bolster dressing inserted.	Marked reduction in swelling; minimal blood loss.
Postoperative period (PICU)	Risk of postoperative airway compromise. Need for ongoing support.	-	Continued intubation, sedation, and mechanical ventilation. ENT wound care. Nasogastric feeds. Broad-spectrum antibiotics continued.	Stable in PICU; gradual clinical improvement.
After microbiology results	Wound and respiratory specimens: heavy growth of MSSA.	Culture and sensitivity: MSSA.	Antibiotics de-escalated based on susceptibility. IV flucloxacillin 50 mg/kg QID commenced on Day 2. Vancomycin + piperacillin-tazobactam discontinued.	Continued clinical improvement on targeted therapy.

Despite the extensive neck swelling, the child remained clinically stable during the initial assessment period, maintaining oxygenation on room air without stridor, drooling, or overt respiratory distress. However, the rapidly progressive bilateral swelling, inability to lie flat, restricted neck movement, and anticipated difficulty in safely obtaining cross-sectional imaging raised concern for potential airway deterioration. After multidisciplinary discussion, elective endotracheal intubation was performed in the PICU about five hours after presentation to secure the airway before CT imaging and subsequent surgical intervention. A difficult-airway trolley was prepared, and intubation was performed using video laryngoscopy in a ramped position with anesthesia and ENT support. The first attempt was successful and hemodynamically uneventful.

Following CT confirmation of extensive disease, he underwent urgent incision and drainage within 30 minutes of CT and six hours after presentation. After the initial incision, a large amount of tense purulent material was released intraoperatively. Approximately 150 mL of purulent fluid with debris was drained. A large left-based submandibular pocket was found to cross the midline through the sublingual space into the right submandibular space. After irrigating the cavity and taking microbiological samples, a bolster dressing and 8-cm Penrose drain were inserted. The external swelling improved dramatically after drainage, and blood loss was minimal.

Postoperatively, the patient remained intubated in the PICU for airway protection and received sedation, mechanical ventilation, nasogastric feeding, wound care, and antimicrobial therapy. Two wound cultures grew MSSA, susceptible to clindamycin, erythromycin, oxacillin, and trimethoprim-sulfamethoxazole. Blood culture showed Gram-positive cocci in clusters; following review by the Infectious Diseases team, this was considered a possible contaminant, and repeat blood cultures were not recommended. Based on the culture and susceptibility results, vancomycin and piperacillin-tazobactam were discontinued, and intravenous flucloxacillin was commenced on Day 2 at 50 mg/kg/dose every six hours.

Outcome and post-discharge follow-up

Following airway control, surgical drainage, and antimicrobial therapy, the patient's clinical condition improved. Sedation was withdrawn, and he was successfully extubated on Day 2, initially requiring nasal cannula oxygen before being weaned to room air without stridor or respiratory distress. Dietitians and speech-language pathologists provided input as feeding was gradually restarted and advanced. The Penrose drain was subsequently removed, and the wound was sutured and managed with bolster dressings and daily ENT review.

During admission, hemoglobin decreased from 8.8 to 7.4 g/dL within 24 hours, and the patient received a blood transfusion. The available PICU documentation did not specify the exact timing, transfusion threshold, or indication. Other inpatient issues included iron-deficiency anemia and the need for nutritional rehabilitation. As recommended by the Infectious Diseases team, he also received catch-up immunizations during admission.

Following six days of intravenous flucloxacillin, therapy was transitioned to oral flucloxacillin 250 mg every six hours on Day 8, as recommended by the Infectious Diseases team, to complete a total antimicrobial course of 14 days from admission. He was subsequently transferred from the PICU to the pediatric ward and discharged home in stable condition on Day 9, with a total hospital stay of 10 days. At discharge, he was afebrile, maintaining oxygen saturation on room air, tolerating oral intake, passing urine and stool normally, and clinically stable. ENT follow-up was arranged, and caregivers were advised to return if symptoms recurred or worsened, breathing became difficult, vomiting recurred, activity or consciousness changed, or oral intake became inadequate.

The patient was reviewed within two days of discharge and remained clinically stable. No immediate recurrence or airway deterioration was documented at this follow-up. The patient did not attend subsequent scheduled outpatient appointments because of reported insurance-related difficulties. The family continued to seek care through the ED as needed; therefore, longer-term structured outpatient follow-up for wound healing, recurrence, feeding status, and completion of antimicrobial therapy was limited.

## Discussion

This case demonstrates several important features of pediatric Ludwig’s angina, a rare but potentially life-threatening infection of the submandibular, sublingual, and submental spaces, with rapid airway compromise being its most serious complication [1-3]. Pediatric cases are uncommon in the literature, although Lin et al. reported that children and adolescents may account for up to one-third of cases reported in the literature [3]. Britt et al. identified only 29 pediatric cases in their review, with a mean age of 9.7 years; they identified an odontogenic source in 54% of cases, while the etiology remained unclear in 27% [2]. Mortality has fallen substantially with modern antibiotics and airway management, from more than 50% in the pre-antibiotic era to approximately 0-10% in contemporary series [2]. In this 26-month-old boy, the Ludwig-space infection was complicated by extensive bilateral abscess formation involving the submandibular, submental, and sublingual spaces. The patient was younger than most reported cases and had a large 9.6 × 4.8 × 6.3 cm abscess containing approximately 150 mL of pus, despite no stridor or drooling.

Comparison with previously reported pediatric cases further demonstrates the severity of the present case. Benhoummad et al. described a two-year-old boy, similar in age to our patient, with rapidly progressive bilateral submandibular, submental, and sublingual infection but no clear dental or systemic source; CT demonstrated extensive disease, and 15 mL of pus was drained, with recovery after broad-spectrum antibiotics [1]. In contrast, our patient had a substantially larger collection, yielding 150 mL of purulent material, and required elective intubation before CT and surgery because of the perceived airway risk. The absence of drooling or stridor in our patient is also noteworthy, as the child remained at significant risk of deterioration despite the lack of these classical airway signs. Pandey et al. similarly emphasized that pediatric Ludwig’s angina presents a potentially difficult airway requiring careful anesthetic planning and experienced multidisciplinary management [4]. Thus, the present case reinforces that airway risk should be assessed based on the overall clinical trajectory and anatomical extent of infection rather than stridor or drooling alone.

Imaging and source control were central to management. Ultrasound initially identified the extensive collection, while contrast-enhanced CT subsequently demonstrated the full extent of deep-space involvement and assisted operative planning. This was followed by urgent incision and drainage, with evacuation of 150 mL of pus. MSSA microbiology allowed antimicrobial therapy to be narrowed to flucloxacillin. Although Ludwig’s angina is frequently associated with odontogenic infection, particularly in older children, no definite source of infection was identified in our patient. Because the oral and dental examination was necessarily limited before airway control, an odontogenic source could not be definitively excluded. The favorable outcome likely reflects early recognition, controlled airway management, appropriate imaging, prompt surgical drainage, and culture-directed antimicrobial therapy.

The child’s unimmunized status is an additional important consideration, although it should not be interpreted as a proven cause or specific risk factor for MSSA Ludwig’s angina. Rather, incomplete immunization is relevant because it leaves young children susceptible to vaccine-preventable invasive infections. This is particularly important for *Haemophilus influenzae* type b, which historically caused serious invasive disease in young children and has rarely been reported in association with Ludwig’s angina. Therefore, the unimmunized status in this case should be viewed primarily as an important preventive health consideration, supporting immunization review and catch-up vaccination, rather than as a direct explanation for the MSSA infection. Overall, this case highlights that pediatric Ludwig’s angina can become extensive and threaten the airway even before classical airway signs appear, emphasizing the importance of early multidisciplinary assessment and intervention [1-4].

## Conclusions

Pediatric Ludwig’s angina can progress rapidly and cause critical airway compromise despite the absence of early signs such as stridor or drooling. This case demonstrates the importance of early recognition of rapidly progressive neck swelling, timely imaging to define the extent of infection, controlled airway management in a monitored setting, prompt surgical drainage, and culture-directed antimicrobial therapy. In this patient, controlled intubation, evacuation of approximately 150 mL of purulent material, and targeted flucloxacillin therapy for MSSA were followed by clinical recovery and discharge in stable condition.
